# What Would Be the Most Appropriate **α**/**β** Ratio in the Setting of Stereotactic Body Radiation Therapy for Early Stage Non-Small Cell Lung Cancer

**DOI:** 10.1155/2013/391021

**Published:** 2013-11-20

**Authors:** Alexander Chi, Sijin Wen, Zhongxing Liao, Jack Fowler, Jiahong Xu, Nam P. Nguyen, James S. Welsh, Ritsuko Komaki

**Affiliations:** ^1^Department of Radiation Oncology, West Virginia University, Medical Center Dr., Morgantown, WV 26506, USA; ^2^Biostatistics Core, Mary Babb Randolph Cancer Center, West Virginia University, Morgantown, WV 26506, USA; ^3^Department of Radiation Oncology, The University of Texas MD Anderson Cancer Center, Houston, TX 77030, USA; ^4^Department of Human Oncology, University of Wisconsin, Madison, WI 53792, USA; ^5^Westat-An Employee-Owned Research Corporation, Rockville, MD 20850, USA; ^6^Department of Radiation Oncology, University of Arizona, Tucson, AZ 85724, USA; ^7^Department of Medical Physics, University of Wisconsin, Madison, WI 53792, USA; ^8^Department of Physics, Northern Illinois University, DeKalb, IL 60115, USA

## Abstract

We hypothesize that the correlation between the radiation dose expressed as the biologically effective dose (BED) and the clinical endpoints will correlate better as the value of the *α*/*β* ratio is increased to >10 Gy, which theoretically minimizes the overestimation of the dose potency associated with the linear quadratic (LQ) formula in the setting of stereotactic body radiation therapy (SBRT) for early stage non-small cell lung cancer (NSCLC). A search was conducted in the PubMed electronic databases in August 2011. In the studies analyzed, increasing the *α*/*β* ratio is associated with an increase in the strength of the correlation between isocenter BED and local control, especially in the studies with median followup of ≥24 months, for which Spearman's correlation coefficients of 0.74–0.76 were achieved for *α*/*β* of 20 Gy, 30 Gy, and 50 Gy (*P* =  0.007–0.008). A trend toward statistical significance was observed for the correlation of isocenter BED and the 2-year overall survival when an *α*/*β* of 20 Gy was used approached statistical significance (*P* = 0.073). Our results suggest that an *α*/*β* > 10 Gy may be more appropriate for the prediction of dose response in the setting of lung SBRT.

## 1. Introduction

Stereotactic body radiation therapy (SBRT), a technique which delivers an ablative dose of radiation over a short period of time, has emerged to become a major noninvasive treatment modality for early stage nonsmall cell lung cancer (NSCLC) worldwide. Excellent local control with tolerable toxicity profile has been consistently reported [1]. In operable patients with stage I NSCLC, long-term overall survival after SBRT may be at least comparable to that after surgery when an adequate dose of radiation was delivered [2]. These excellent clinical outcomes provide evidence for SBRT's increasing role in the treatment of early stage NSCLC as an alternative to surgery.

Due to the large variation in the dose fractionation schedules used in clinical practice, dose response following SBRT was investigated and demonstrated after the radiation dose delivered is converted to the biologically effective dose (BED) after linear quadratic normalization [3, 4]. The BED, the total dose which could cause the same log cell kill as a specific dose fractionation schedule under consideration if it is delivered in infinitely small fractions well spaced out or at an infinitely low-dose rate, is a mathematical term derived from linear quadratic cell survival outcome in radiobiology [5]. Because of the short overall treatment time (usually <2 weeks), the overall treatment time factor is often ignored in the BED calculation when SBRT was delivered for early stage NSCLC [3–5].

Despite this wide adaptation of the BED in lung SBRT, many have questioned its validity because of the potential overestimation of tumor cell kill when large doses of radiation are delivered in 1 fraction by the linear quadratic (LQ) formalism [6–8]. To avoid overpredicting the potency and toxicity of SBRT, a new mathematical model, the universal survival curve (USC), has been created to better fit the NSCLC cell survival curve [9]. This model was created by adopting the multitarget model of tumor cell kill (a model which predicts cell survival by assuming that cell kill depends on whether a number of critical targets within a cell are hit after irradiation) into the LQ formalism at a certain radiation dose level, the transition dose, because the multitarget model has been shown to fit empirical cell survival data well in the high-dose range. Although being very sophisticated, the complexity of this model poses a challenge to the clinician in day to day clinical practice. In addition, there are a number of parameters which need to be defined and validated prior to further clinical adaptation. On the contrary, the LQ model has been used for decades in the radiation oncology community and is widely adapted to clinical use. Therefore, simpler approaches to minimizing the overestimation of tumor cell kill after ablative doses of radiation through the LQ model should be sought.

As shown previously, NSCLC cells can possibly repopulate as fast as oropharyngeal cancer cells [10]. It has been observed that rapidly repopulating tumors are likely to have a greater *α*/*β* ratio, which is approximately 20 Gy for oropharyngeal cancers [11]. Therefore, a tumor *α*/*β* ratio of higher than 10 Gy may be more appropriate for NSCLC. Higher *α*/*β* ratio has been known to produce straighter curves in the high-dose region through the LQ model. Therefore, the potential overestimation of dose response in high-dose regions by the LQ model in the setting of SBRT for early stage NSCLC may be reduced by simply using an *α*/*β* ratio of higher than 10 Gy when calculating tumor BED, especially when large fractional doses are used [12]. In the current study, we investigate the dose response relationship between the tumor isocenter BED and local tumor control following SBRT for early stage NSCLC and explore which *α*/*β* ratio is the most appropriate for tumor BED calculation in this setting.

## 2. Materials and Methods

### 2.1. Search Strategy

This systematic review was designed to explore what *α*/*β* ratio should be most appropriate for BED calculation in the setting of SBRT for early stage NSCLC. This is assessed by investigating the strength of correlation between the tumor BED at the isocenter of the target calculated with various randomly selected *α*/*β* ratios (5, 8.2, 10, 20, 30, and 50 Gy) and the clinical endpoints of local tumor control and overall survival. A search based on PubMed electronic databases was conducted to select studies outlining the following: local control for early stage NSCLC following SBRT; toxicity following SBRT for NSCLC. The following terms were explored and used for each database search: non-small cell lung cancer, early stage, stage I, stereotactic body radiotherapy, stereotactic radiosurgery, and stereotactic ablative radiotherapy. Reference lists of relevant papers were then searched for additional publications. The tumor BED was calculated based on the linear quadratic formula with *α*/*β* ratios described above. For this study, the tumor BED is the BED at the isocenter of the target volume. Local control refers to the rate of tumor control at the primary site only for the duration of each study. It equals the number of patients with the tumor controlled locally decided by the total number of patients treated for a specific dose fractionation schedule.

### 2.2. Statistical Analysis

Scatterplots and nonparametric regression lines are generated using lowess smoothers [13, 14] to illustrate the relationship between radiation dose and clinical endpoints. A nonparametric Spearman's rank correlation coefficient [15] was calculated to estimate the degree of correlation between the radiation dose, which is expressed as the tumor BED at the target volume's isocenter, and the local control and the 2-year overall survival rates obtained from different studies, assuming that the relationship between the tumor BED and these clinical endpoints can be described by a nonlinear monotonic function.

## 3. Results

### 3.1. Quantity of Studies

A total of 24 studies reporting the clinical outcome associated with one dose fractionation scheme for which the isocenter dose can be calculated (most commonly used scheme if outcome following multiple dose fractionation schemes was reported) following SBRT delivered with photon therapy for stage I, and mostly T1, NSCLC were identified [16–39]. They were found through the PubMed electronic database searches after exclusion of duplicate, irrelevant references, as well as abstracts. All studies were published between 2003 and 2011. The studies used for the current analysis include most retrospective and a few prospective studies [16, 20–22, 24, 26, 35]. The most commonly used dose fractionation schedule, the number of patients treated with the listed dose fractionation schedule/the total number of patients underwent SBRT reported in each study, median follow-up time in months for each study, the associated local tumor control from each study, the dose calculation algorithm, and whether image guidance was used for radiation delivery are summarized in Table 1. The use of IGRT in the study by Fakiris et al. [16] was implied from a previous publication [40]. For the purpose of this study, studies of no more than 10 patients and the studies in which no description of the local tumor control for a specific dose fractionation regimen can be obtained in the actual publication or through communication with the corresponding authors were excluded. Also, studies that were published only in abstract form were excluded. For studies from the same group of investigators, only the latest publication detailing the specific dose fractionation regimen and corresponding local tumor control was included.

### 3.2. Correlation of Fractional Dose and Local Control

**Figure 1:**
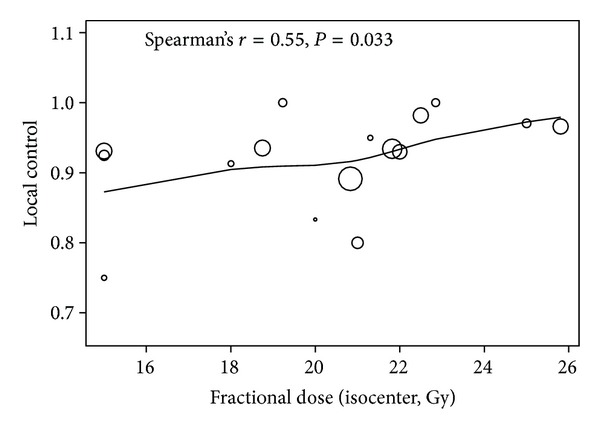
The correlation between the fractional dose and the local control in studies which reported the outcome of dose fractionation schedules that delivered the total dose in 3 fractions. The size of the circle is proportional to the number of patients treated with a specific dose fractionation schedule in each study.

As shown in Table 1, a 3-fraction schedule delivering fractional doses from 15 Gy to 25.8 Gy to the tumor isocenter was used in 16/24 studies. A positive dose response between the fractional dose and local control among these studies has been observed with a Spearman's rank correlation coefficient of 0.55 (*P* = 0.033). This dose response relationship is shown in Figure 1. This dose response relationship was not observed in studies that used ≥4 fractions most likely due to the small number of studies.

### 3.3. Correlation of Isocenter Tumor BED and Local Control

**Figure 2:**
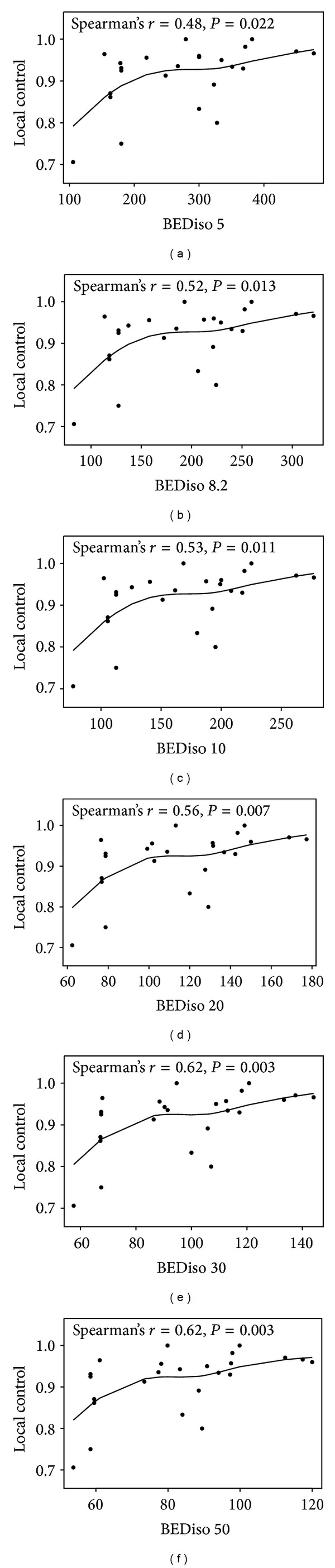
The correlation of the tumor BED at the isocenter calculated with a spectrum of *α*/*β* ratios (5, 8.2, 10, 20, 30, and 50 Gy) and the local control reported among all studies. Due to the use of different *α*/*β* ratios, the BED scale on the *x*-axis is different for each plot.

After the isocenter BEDs were calculated with gradually increasing but randomly selected values of *α*/*β* ratio through the linear quadratic formula, they were correlated with the associated local control values. As shown in Figure 2, increased isocenter BED correlated with an increase in local control regardless of which *α*/*β* ratio was used in the BED calculation. The isocenter BEDs calculated with *α*/*β* ratios ranging from 5 to 50 Gy were all significantly correlated with the local control. However, the Spearman's rank correlation coefficient appeared to increase more noticeably as the value of *α*/*β* ratio increased to over 10 Gy. The strength of correlation (statistical significance) between tumor isocenter BED and the local control also increased as the *α*/*β* ratio increased, especially when an *α*/*β* ratio of >10 Gy was used.

**Figure 3:**
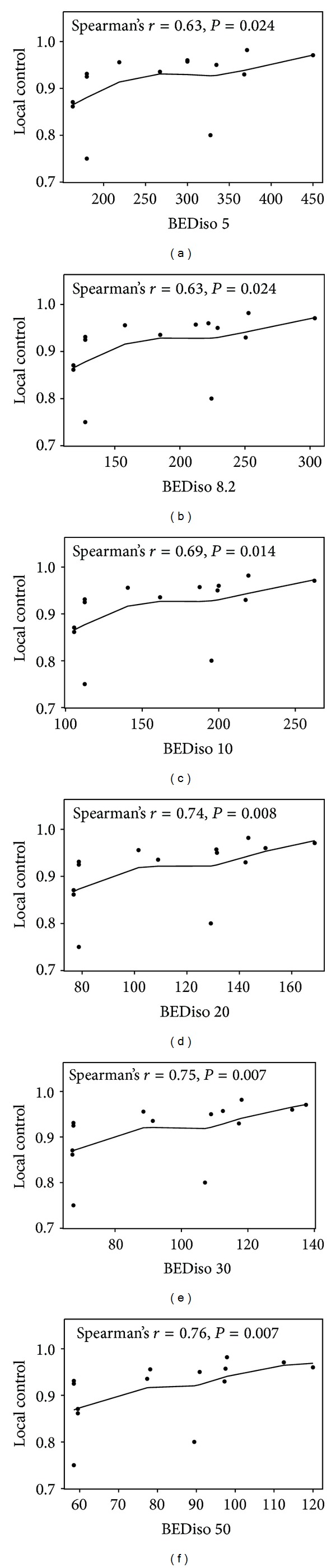
The correlation of the tumor BED at the isocenter calculated with a spectrum of *α*/*β* ratios (5, 8.2, 10, 20, 30, and 50 Gy) and the local control reported among studies with a median followup of ≥24 months. Due to the use of different *α*/*β* ratios, the BED scale on the *x*-axis is different for each plot.

When studies with median followup of at least 24 months were analyzed separately [16–29], correlation between the isocenter BED calculated with the same set of *α*/*β* ratios and the local control rate reported in these studies was also observed. As shown in Figure 3, a trend toward stronger correlation between isocenter BED and local control is observed as higher *α*/*β* ratio is used in BED calculation. The highest correlation is observed for *α*/*β* ratios of 20, 30, and 50 Gy with correlation coefficients of 0.74–0.76 (Figure 3).

### 3.4. Correlation of Isocenter Tumor BED with 2-Year Overall Survival

**Figure 4:**
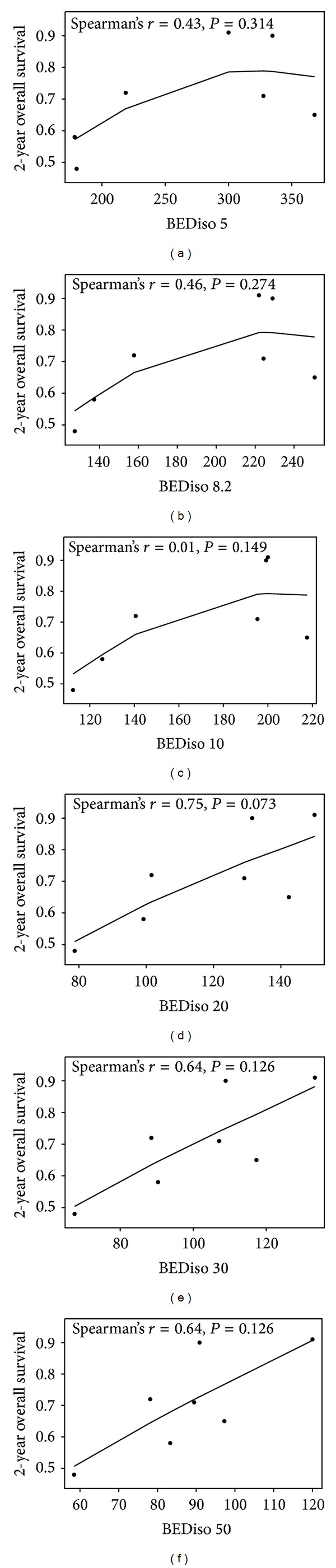
The correlation of the tumor BED at the isocenter calculated with a spectrum of *α*/*β* ratios (5, 8.2, 10, 20, 30, and 50 Gy) and the 2-year overall survival reported in selected studies. Due to the use of different *α*/*β* ratios, the BED scale on the *x*-axis is different for each plot.

Seven of the 24 studies reported overall survival (OS) at 2 years [18–20, 24, 27, 29, 39]. Among all the survival endpoints reported, including disease-free survival, cause-specific survival, and overall survival (OS), only OS at 2 years [18–20, 24, 27, 29, 39] demonstrated a positive correlation with radiation dose described as the tumor BED at the treatment isocenter. This is shown in Figure 4. The positive correlation is most noticeable when the isocenter BED is calculated with *α*/*β* ratios of >10 Gy. The strongest correlation was observed when the isocenter BED is calculated with an *α*/*β* ratio of 20 Gy, and this correlation approached statistical significance with a Spearman's rank correlation coefficient of 0.75 (*P* = 0.073). 

## 4. Discussion

As a tissue specific fractionation sensitivity parameter in the LQ formula, the *α*/*β* ratio has commonly been accepted to be approximately 10 Gy for tumor or “early responding” tissue. However, more studies are reporting a highly variable range of *α*/*β* ratios for various cancers based on individual tumor's rate of proliferation [10, 11, 41, 42]. “Individualization” of *α*/*β* ratios based on specific tumor type may be important in the setting of SBRT, in which the LQ model has been criticized for overpredicting dose potency when large fractional dose, such as 20 Gy, is delivered [9]. This is because of the fact that the overestimation of radiation efficacy can be effectively reduced if the *α*/*β* ratio is increased to 20 Gy to account for the rapidly proliferating nature of NSCLC cells when SBRT is used to deliver large fractions over ≤5 fractions [12]. On the other hand, some have found that the LQ model may underestimate the tumor control after stereotactic radiosurgery intracranially, which may be caused by indirect tumor cell killing triggered by significant vascular damage after ablative doses are delivered [43]. However, this concept needs to be further explored in future studies on NSCLC. 

The most important finding of this study is that the biologically effective dose response relationship in the setting of lung SBRT appears to be very stable throughout a spectrum of *α*/*β* ratios. This implies the robustness of the linear-quadratic (LQ) formalism in predicting dose response and its clinical applicability in the setting of lung SBRT. The correlation between local control and the isocenter BED increased when the *α*/*β* ratio used in BED calculation increased from 5 Gy to 50 Gy (Figures 2 and 3). The best correlation was observed when studies with at least a median followup of 24 months were analyzed separately (Figure 3). These studies were analyzed separately because longer followup usually brings more stability and reliability to the results reported. Thus, the clinical evidence suggests that utilizing an *α*/*β* ratio of >10 Gy in the LQ formalism may lead to more accurate description of the biologically effective dose response relationship in the setting of SBRT for early stage NSCLC. Among all the survival data endpoints, the OS at 2 years among 7 studies also demonstrated a correlation with the BED. Within the spectrum of *α*/*β* ratios tested, this correlation was the strongest with a Spearman's correlation coefficient of 0.75 when an *α*/*β* ratio of 20 Gy was used for BED calculation (Figure 4). This correlation reached marginal statistical significance (*P* = 0.073). This finding supports the use of higher *α*/*β* ratios, such as 20 Gy, for the prediction of biologically effective dose response in the setting of lung SBRT. For an *α*/*β* ratio of infinity, the Spearman's correlation coefficient was 0.40 (*P* = 0.15) for correlation between local control and tumor BED in studies with median followup of at least 24 months. Similarly, there was no statistically significant correlation when physical dose was used to correlate with 2-year OS (*ϱ* = 0.02; *P* = 0.99). This suggests that the physical dose should not be used directly for dose response estimation in the setting of lung SBRT.

The robustness of the LQ model in the prediction of biologically effective dose response observed in our study is also supported by other studies [44, 45]. The value of the *α*/*β* ratio to be used for iso-dose effect comparison of the SBRT dose fractionation schedules and conventionally fractionated dose regimens has been estimated to be 8.2 Gy in a modeling study by Stuschke and Pöttgen [44]. In another modeling study by Partridge et al., the dose response relationship between the tumor BED and the disease-free survival (DFS) for stage I–III NSCLC treated with both conventionally fractionated radiotherapy and SBRT has been explored [45]. The fitting of their dose response model appears to be very stable with *α*/*β* ranging from 8 Gy to 14 Gy. Our study is different from the previous studies in that it further suggests that an *α*/*β* of >10 Gy may be comparatively more appropriate in the setting of lung SBRT even that spectra of *α*/*β* ratios from 5 Gy to 50 Gy may all be valid. However, this should be further validated in a large prospective study to include NSCLC patients who are treated with both conventionally fractionated radiotherapy and SBRT. This will need to be conducted to further investigate if a single *α*/*β* that would be suitable for both conventionally fractionated radiotherapy and SBRT can be defined. Furthermore, our study analyzes a relatively more homogeneous group of studies which report the clinical outcome following SBRT only. This minimizes the confounding factors, such as the delayed tumor cell repopulation associated with conventionally fractionated radiotherapy, the difference in reassortment, and reoxygenation between conventionally fractionated radiotherapy and ablative radiotherapy, as well as differences in treatment outcome related to the different technology used for radiotherapy delivery.

Spearman's correlation coefficient was used to assess the relationship between the tumor BED and the clinical endpoints assuming a nonlinear monotonic function. However, the study size was not taken into account when Spearman's correlation coefficient was calculated, which is a major limitation of our study. As a result, the findings from this study can only be suggestive and need to be further validated in future studies. Also, the heterogeneous nature of the different patient populations included in different studies greatly limits the study and warrants future investigation in a study that will assess tumor BED and clinical outcome based on individual patient outcome. Another limitation is that there were only 7 studies reporting the 2-year overall survival, which may be too few to establish a correlation with a high level of confidence.

## 5. Conclusion

Our study demonstrates that increasing the *α*/*β* ratio is associated with an increasing trend in the strength of the biologically effective dose response relationship, especially when the *α*/*β* ratio is increased to ≥20 Gy. All of our findings support the previous hypothesis that the overestimation of tumor cell kill from the LQ model may be reduced by simply increasing the value of *α*/*β* [12]. However, our study is limited by the heterogeneous nature of different studies. As a result, this study cannot provide a definite *α*/*β* ratio in the setting of SBRT for early stage NSCLC. But it does suggest that an *α*/*β* > 10 Gy may be more appropriate for the prediction of dose response in the setting of lung SBRT.

## Figures and Tables

**Table 1:** Local tumor control following SBRT for early stage NSCLC.

References	No. of fractions	No. of patients/no. of total patients	Fractional dose (isocenter, Gy)	Median followup (months)	Local control	Dose calculation algorithm
Kopek et al. [17]	3	62/88	15.0	44.0	93.1%	PB^¶^
Koto et al. [22]		20/31	15.0	32.0	75.0%	Batho^¶^
Hoyer et al. [24]		40/40	15.0	29.0	92.5%	NSS^¶^
Bral et al. [35]		23/40	18.0	16.0	91.3%	NSS, BrainLab^¶^
Ricardi et al. [26]		62/62	18.8	28.0	93.6%	CC^¶^
Guckenberger et al. [37]		32/124	19.2	14.0	100.0%	CC^¶^
Ng et al. [30]		12/20	20.0	21.0	83.3%	NSS, BrainLab^¶^
Andratschke et al. [31]		92/92	20.8*	21.0	89.1%	NSS^¶^
Nyman et al. [18]		45/45	21.0	43.0	80.0%	Batho^¶^
Vahdat et al. [19]		20/20	21.3*	43.0	95.0%	NSS, Cyberknife^¶^
Crabtree et al. [33]		76/76	21.8	19.0	93.4%	NSS, Triology^¶^
Baumann et al. [20]		57/57	22.0	35.0	93.0%	PB^¶^
Timmerman et al. [21]		59/59	22.5	34.4	98.2%	NSS^¶^
Turzer et al. [38]		31/36	22.9	13.8	100.0%	CC^¶^
Fakiris et al. [16]		34/70	25.0	50.2	97.1%	NSS^¶^
van der Voort van Zyp et al. [36]		59/70	25.8	15.0	96.6%	NSS, Cyberknife^¶^
Matsuo et al. [23]	4	101/101	12	31.4	86.1%	PB
Baba et al. [28]		85/124	12	26.0	87.1%	NSS
Stephans et al. [32]	5	56/86	10.2	19.8	96.4%	NSS, BrainLab^¶^
Takeda et al. [29]		63/63	12.5	24.0	95.6%	SC
Lagerwaard et al. [25]		>100^‡^/—	15.0	29.0	95.7%	NSS, BrainLab^¶^
Onimaru et al. [34]	8 to 10	17/45	6.0	18.0	70.6%	PB & SC^¶^
Onishi et al. [39]		35/35	7.3	13.0	94.3%	NSS^¶^
Xia et al. [27]		43/43	10.0	27.0	96.0%	NSS, Gamma knife

*Median or mean dose; ^‡^Personal communication with Dr. Lagerwaard.  ^¶^Image guidance applied. CC: Collapsed cone; NSS: not specifically stated; PB: pencil beam; S: superposition.
